# Colistin Resistance Among Multi-Drug Resistant Gram-Negative Bacterial Isolates From Different Clinical Samples of ICU Patients: Prevalence and Clinical Outcomes

**DOI:** 10.7759/cureus.28317

**Published:** 2022-08-23

**Authors:** Kumudini Panigrahi, Basanti K Pathi, Nirmala Poddar, Smaranita Sabat, Sujit Pradhan, Dipti Pattnaik, Shubhransu Patro, Ashok K Praharaj

**Affiliations:** 1 Microbiology, Kalinga Institute of Medical Sciences, Bhubaneswar, IND; 2 Community Medicine, Institute of Medical Sciences and SUM Hospital, Bhubaneswar, IND; 3 Infectious Diseases, Kalinga Institute of Medical Sciences, Bhubaneswar, IND; 4 General Medicine, Kalinga Institute of Medical Sciences, Bhubaneswar, IND

**Keywords:** gram negative bacilli, clinical outcome, prevalence, colistin, multi drug resistance

## Abstract

Introduction: Colistin is considered to be the last resort for the management of infections caused by multi-drug resistant (MDR) gram-negative bacilli (GNB). However, in the recent past, there has been a rise in colistin resistance among MDR isolates in clinical settings with no profound data on the incidences and causes. The purpose of this study was to estimate the prevalence of colistin-resistance (CLR) in MDR isolates collected from different intensive care units (ICUs) and to determine the clinical outcomes of the patients.

Materials and methods: A prospective study was conducted in the ICU of a tertiary care hospital in Eastern Odisha, India from March 2019 to February 2020. MDR GNB isolates from different clinical samples of ICU patients, not intrinsically resistant to colistin, were included in this study. Samples collected for culture and sensitivity testing were processed as per standard guidelines in the microbiology laboratory. MDR organisms were examined for colistin susceptibility by the broth dilution method. Clinical data was collected from hospital electronic medical records and presented as percentage, number (N), and median (range).

Results: The prevalence of colistin resistance MDR GNB was found to be 19.6% in the present study. Colistin resistance among the MDR isolates was found to be the highest (9.2% for *Klebsiella pneumonia *followed by 5% for *Escherichia coli*). CLR drug-resistant isolates were commonly (28.8%) isolated from samples of respiratory tract infections and the majority (54.1%) were from neurology ICU. In this study, co-morbidity was not found among 57.9% of the ICU patients and recovery was maximum i.e., 74.2%.

Conclusion: This study found the prevalence of colistin resistance to be high (19.6%) among all MDR GNB isolates from samples of ICU patients, *Klebsiella pneumonia* and *Escherichia coli* commonly acquire colistin resistance. Patients in the neurology ICU were frequently infected with CLR MDR strains. Most of the patients who recovered were without any underlying comorbidities. Prolonged hospital stay and direct antibiotic pressure in the hospital can lead to the development of CLR variants.

## Introduction

The patients admitted to the intensive care units (ICU) are more prone to nosocomial infections which is the prime cause of mortality [1,2]. In a study, Cohen (2013) reported an extended prevalence of ICU infections in 51% of patients with gram-negative pathogens [2-3]. Anti-microbial resistance is an increasing concern in the treatment of nosocomial infections particularly in ICU patients [3].

In the case of multi-drug resistant (MDR) gram-negative bacilli (GNB) infection, colistin is considered to be one of the commonly used antibiotics of choice for treatment. This antibiotic has been used in veterinary science since the mid-twentieth century [4]. The use of this drug in humans has been restricted for a decade due to its known toxicity, except in the case of decontamination regimens in the ICU and nebulized administration in cystic fibrosis [4,5]. The use of this molecule was resumed to solve the therapeutic challenges that emerge due to carbapenem-resistant GNB infection. However, many unresolved issues like pharmacokinetics in critically ill subjects, dosing, nephrotoxicity, paucity of susceptibility data, and development of resistance limited their use [4-7]. Hence, there is a lack of consensus on the optimum use of this antibiotic to date [7].

Colistin, a cyclic polypeptide antibiotic, breaks the outer cell membrane of most of the GNB by interacting with the lipopolysaccharide (LPS) [8]. Colistin resistance is sporadic in GNB, except in those with intrinsic resistance [9-12]. The acquisition of colistin resistance in *Klebsiella pneumoniae*, *Acinetobacter baumannii*, and *Pseudomonas aeruginosa* is rarely reported [13,14]. To date, the acquired colistin resistance in the bacterium is thought to be due to chromosomal mutations in the gene that are associated with LPS alteration [8]. Recently, mcr-1 and mcr-2, the two plasmid-mediated CLR genes are believed to contribute to colistin resistance [15-18]. However, there is a lack of systematic study to find out the prevalence of CLR in MDR GNB in clinical samples collected from ICU patients. A few published reports pertaining to this issue were a part of the antimicrobial surveillance plan where the strains were selected randomly [9-12]. The first report pertaining to colistin resistance in GNB isolates was published in the year 2013. However, the report was not representative of the whole population due to a smaller sample size. In all GNB isolates from clinical samples, colistin is not regularly checked and that may lead to unawareness of resistance.

The purpose of this single-center study was to estimate the prevalence of colistin-resistance (CLR) in MDR GNB isolated from patients in different intensive care units and to determine their clinical outcomes.

## Materials and methods

This was a prospective study conducted in the ICU of a tertiary care hospital in Eastern Odisha, India after approval from the Institutional Ethical Committee of the hospital (KIMS/KIIT/IEC/52/2018) from March 2019 to February 2020. MDR GNB isolates from different clinical samples of ICU patients, not intrinsically resistant to colistin, were included in this study. Patients with the growth of organisms other than MDR GNB and organisms with intrinsic resistance to colistin were excluded from this study.

Samples from ICU patients, after proper collection in appropriate sterile containers, were sent to the microbiology laboratory for culture and sensitivity testing. Different samples were collected and processed in the laboratory based on the standard microbiology procedure. Samples like sputum, endotracheal aspirate, broncho alveolar lavage fluid, and pus were inoculated into solid media (blood agar and MacConkey agar) and liquid media (Tryptone-Soy Broth); urine samples were inoculated into cystine lactose electrolyte deficient (CLED) agar medium. Blood and body fluids were inoculated in BacT/ALERT (bioMérieux, Inc., Durham, NC) broth bottle and incubated at 37ºC. Vitek-2 compact identification system (bioMérieux, France) was used to process and identify any significant growth that was obtained. Antibiotic susceptibility testing of the isolates was done by the Vitek-2 antibiotic susceptibility testing (AST) system as part of routine microbiology, according to the Clinical and Laboratory Standards Institute (CLSI) guidelines. Antibiotic panels tested for significant gram-negative isolates were ampicillin, amoxyclav, amikacin, ciprofloxacin, ceftriaxone, cefuroxime, cefepime, ertapenem, nitrofurantoin, gentamicin, imipenem, meropenem, nalidixic acid, cefoperazone-sulbactam, cotrimoxazole, tigecycline, piperacillin-tazobactam. The organisms were marked as susceptible or resistant (intermediately susceptible were also considered as resistant) to any of these antibiotics as per minimum inhibitory concentration (MIC) value and CLSI guidelines. MDR was defined as acquired non-susceptibility to at least one agent in three or more antimicrobial categories [19-20]. 

MDR GNB were selected and tested for colistin susceptibility by broth microdilution test with colistin sulphate powder (Hi Media Laboratory, Mumbai, India), since methods like Kirby-Bauer disc diffusion test, E-test, or Vitek-AST are not currently acceptable for colistin, as per EUCAST (European Committee for Antimicrobial Susceptibility Testing) and CLSI subcommittee CLR guidelines [21]; isolates were marked as susceptible or resistant to colistin as per the guidelines [21].

For the collection of relevant clinical data, the electronic medical records of the patients were referred. Data was available for 240 patients from the repository. Outcomes of the group of the patients infected with CLR GNB were found in terms of clinical recovery or death of the patient.

The demographic data like age and sex were mentioned as median and percentage. Other relevant data were presented as percentage, number (N), and median (range). The complete statistical analysis was performed by SPSS statistical package, version 25.0 (IBM Corp., Armonk, NY).

## Results

In the present study, out of 357 MDR GNB isolates, a total of 70 CLR isolates of acquired type were identified which corresponds to an overall prevalence of 19.6% (Table 1). Colistin resistance was found in 33/357 isolates of *Klebsiella pneumoniae* with a 9.2% prevalence. Besides, the resistance was also detected in 18/357 isolates of *Escherichia coli*, 5/357 isolates of *Pseudomonas aeruginosa, *and 10/357 isolates of *Acinetobacter baumannii* with a prevalence of 5%, 1.4%, and 2.8% respectively. The other CLR GNB details are presented in Table 1. The range of colistin MICs was 0.5-16 µg/ml except in *Acinetobacter lwoffii* where the MIC was 16 µg/ml. The MDR GNB isolates were recovered mostly 98/357 (26.8%) from blood cultures followed by ulcer/surgical wound exudates (23.0%) and urine cultures (22.7%).

**Table 1:** Prevalence of colistin resistance among multi-drug resistant (MDR) gram-negative bacteriae isolated from samples of ICU patients MIC-Minimum inhibotory concentration, microgram/millilitre

Organisms	Klebsiella pneumoniae (n= 33)	Escherichia coli (n=18)	Pseudomonas aeruginosa (n=5)	Acinetobacter baumannii (n=10)	Klebsiella oxytoca (n=0)	Citrobacter freundii (n=2)	Acinetobacter lwoffii (n=1)	Enterobacter aerogenes (n=0)	Unidentified (n=1)	Pantoe species (n=0)	Enterobacter cloacae (n=0)	Citrobacter amalonaticus (n=0)	Pseudomonas species (n=0)	Total (n=70)
Prevalence of colistin resistance, %	9.2%	5.0%	1.4%	2.8%	0.0%	0.6%	0.3%	0.0%	0.3%	0.0%	0.0%	0.0%	0.0%	19.6%
Colistin MIC range, µg/ml	0.5-16	0.5-16	0.5-16	0.5-16	0.5-16	0.5-16	16	0.5-16	0.5-16	0.5-16	0.5-16		0.5-16	
Multidrug resistance (n ) MDR	166	89	32	49	5	4	1	3	3	2	1	1	1	357
Pus	1	1	0	1	0	0	0	0	0	0	0	0	0	3 (0.8%)
Urine	31	30	6	14	1	0	0	0	0	1	0	0	0	83 (22.7%)
Blood	50	19	10	12	2	2	0	1	2	0	0	0	0	98 (26.8%)
Sputum	19	9	3	4	0	1	0	0	0	0	0	1	0	37 (10.1%)
Exudate	40	19	8	11	1	1	1	1	0	1	0	0	1	84 (23.0%)
Others	25	11	5	7	1	0	0	1	1	0	1	0	0	52 (14.5%)
Total	166	89	32	49	5	4	1	3	3	2	1	1	1	357 (100%)

The clinical and sociodemographic data like age and sex are presented in Table 2. The median age of the patients were found to be 54.81 ± 21.5 years (range 1-94). Majority of the population were male (n = 226, 65.9%). Out of 240 data available from the repository, most of the patients presented with respiratory tract infection (31.4%) followed by septicemia (25%) and urinary tract infection (14.3%). Majority of CLR MDR isolates were from specimens of respiratory tract infections i.e., 22/52 (42.3%) followed by blood from septicemia i.e., 12/52 (23.0%).

**Table 2:** Distribution of age, sex and clinical characteristics of patients with multi-drug resistant (MDR) gram-negative bacterial isolates and colistin-resistant MDR gram-negative bacterial isolates

Characteristics	Values for patients with multidrug resistant gram negative bacterial isolates	Values for patients with colistin resistant multidrug resistant gram negative bacterial isolates
Median Age, (range) in years	54.81 (1 – 94)	54.81 (1 – 94)
Males (n, %)	235 (65.9%)	51 (72.8%)
Females (n, %)	122 (34.1 %)	19 (27.2%)
Clinical presentation	n = 240 (%)	n = 52 (%)
Septicaemia	60 (25.0)	12 /52 (23.0 %)
Respiratory Tract infection	76 (31.4)	22/ 52 (42.3 %)
Urinary Tract infection	34 (14.3)	7/ 52 (13.4 % )
Meningitis and other neurological infections	15 (6.5 %)	3/52 (5.7 %)
Others	37 (15.8 %)	8 /52 (15.3 %)

The most prevalent comorbidity was chronic kidney disease with diabetes i.e., 24/240 (10%). In this study, out of the total patient data collected, 57.9% of the patients had no comorbidity; the majority of 55% of them recovered. Recovery was a maximum 139/240 (74.2%) among the patients with no underlying comorbidity. There were two patients with the diagnosis of brain tumor and both of them died. Patients of diabetes with underlying kidney disease were 24 in number and 83.4% of them died (Table 3).

**Table 3:** Distribution of clinical outcomes of the patients with their co-morbidity status

Clinical outcomes		Comorbidities													Total
		Nil	Chronic kidney disease	Chronic kidney disease+ Diabetes	Diabetes	Head Injury	Brain Tumor	Hemiplegia	Post Operative infection	Preterm baby	Subdural Hematoma	Pneumonia , Ventilated patients	Cancer prostate	Metastatic cancer	
Died	Count	41	3	20	16	0	2	8	4	2	0	7	3	2	108
	% of Total	37.9 %	2.7 %	18.5 %	14.8 %	0.0%	1.8%	7.4 %	3.7 %	1.8%	0.0%	6.4 %	2.7 %	1.8%	45 %
Recovered	Count	98	3	4/24	4	9	0	4	4	2	2	2	0	0	132
	% of Total	74.2 %	2.27 %	3.03 %	3.03 %	6.8 %	0.0%	3.03 %	3.03 %	1.5%	1.5%	0.5%	0.0%	0.0%	55 %
Total	Count	139	6	24	20	9	2	12	8	4	2	9	3	2	240
	% of Total	57.9 %	2.5%	10 %	8.3 %	3.7 %	0.8%	5 %	3.3%	1.6%	0.8 %	3.7 %	1.2%	0.8%	100.0%

In this study, the majority of CLR MDR strains were isolated from neurology ICU patients i.e., 46/70 (65%) followed by patients of neonatal ICU, i.e., 11/70 (15.7%) (Table 4).

**Table 4:** ICU-wise distribution of multi-drug resistant (MDR) gram-negative bacterial isolates and colistin resistant (CLR) MDR gram-negative bacteria isolates

Organisms		Location					Total
		Pediatrics ICU	Neurology ICU	Cardiac ICU	Medicine ICU	Neonatal ICU	
Klebsiella pneumoniae	Number of MDR Strains	8	106	1	27	24	166
	Number of CLR MDR strains	1	23	0	4	5	33
Escherichia coli	Number of MDR Strains	5	42	0	27	15	89
	Number of CLR MDR starins	2	11	0	2	3	18
Pseudomonas aeruginosa	Number of MDR Strains	3	15	0	9	5	32
	Number of CLR MDR starins	0	4	0	1	0	5
Acinetobacter baumannii	Number of MDR Strains	2	22	1	12	13	49
	Number of CLR MDR starins	1	5	0	2	2	10
Klebsiella oxytoca	Number of MDR Strains	0	3	0	1	1	5
	Number of CLR MDR starins	0	0	0	0	0	0
Citrobacter freundii	Number of MDR Strains	1	2	0	1	0	4
	Number of CLR MDR starins	0	2	0	0	0	2
Acinetobacter lwoffii	Number of MDR Strains	0	1	0	0	0	1
	Number of CLR MDR starins	0	1	0	0	0	1
Enterobacter aerogenes	Number of MDR Strains	0	1	0	1	1	3
	Number of CLR MDR starins	0	0	0	0	0	0
Unidentified	Number of MDR Strains	1	2	0	0	0	3
	Number of CLR MDR starins	0	0	0	1	0	1
Pantoea species	Number of MDR Strains	0	0	0	1	1	2
	Number of CLR MDR starins	0	0	0	0	0	0
Enterobacter cloacae	Number of MDR Strains	0	1	0	0	0	1
	Number of CLR MDR starins	0	0	0	0	0	0
Citrobacter amalonaticus	Number of MDR Strains	0	1	0	0	0	1
	Number of CLR MDR starins	0	0	0	0	0	0
Pseudomonas species	Number of MDR Strains	0	0	0	0	1	1
	Number of CLR MDR starins	0	0	0	0	0	0
Total	Number of MDR Strains	20 (5.6%)	194 (54.3%)	2 (0.56%)	79 (22.12%)	62 (17.36%)	357 (100%)
	Number of CLR MDR starins	4 (5.7%)	46 (65%)	0 (%)	9 (12.8%)	11(15.7%)	70 (100%)

## Discussion

Colistin is one of the commonly used antibiotics in infections caused due to MDR organisms. Presently, the emergence of plasmid-mediated CLR in the GNB has greatly limited its use against many aggressive infections [22]. Hence, the rationale of the present study was to estimate the prevalence of CLR in MDR GNB isolated from patients admitted in different ICUs of a tertiary care health centre and to determine the clinical outcomes of the patients

The highlight of this study is the high prevalence of CLR in MDR GNB infections and its association with clinical outcomes. According to a study from India [16], the overall prevalence of CLR (19.6%) of acquired type was much higher than the reported prevalence in western countries [8,15]. For the high prevalence of CLR among MDR isolates in our study, several factors are taken into consideration. There is frequent use of colistin, as it is not very tightly regulated till now, and in infections still susceptible to lower antibiotics, it is used occasionally in suboptimal doses. Colistin is frequently used in farm and dairy animals, in agriculture, and in pisciculture which leads to the leaching of colistin into the environment in low doses and induction of CLR among saprophytic organisms, which later enters the human body in different ways [17].

In our study, the rate of CLR was found to be more in *Klebsiella pneumoniae* and *Escherichia coli* as compared to other species which is in agreement with a study done by Zafer et al. [23]. Similarly, the present findings are consistent with previously published studies [24-27]. As per previous findings, the incidence of resistant subpopulation among hetero-resistant GNB-like members of the *Enterobacteriaceae *family may augment due to colistin treatment [28]. Besides, the same study suggests that infections triggered by these organisms may have a negative clinical outcome following colistin administration due to cross-resistance to host the innate immune system [28]. Recent global antimicrobial surveillance programme reports have also enumerated a low prevalence of CLR among Enterobacteriaceae isolates [11,12]. Some studies mention the augmentation of the CLR rate among carbapenemase producers [12]. As CLR is not routinely tested, the exact extent of acquired CLR is not yet known. In this study, the prevalence of CLR among MDR *Pseudomonas aeruginosa *is 1.4% and among *Acinetobacter spp. *is 2.8%, which is much less in comparison to other studies. In a surveillance of MDR organisms in a tertiary health care setting in North India, Wattal et al. found 8% of *Pseudomonas spp.* to be resistant to colistin in ICU samples [7]. In 2011, a multicentric study from Kuwait pointed to a relatively high rate of CLR of 12% among the 250 *Acinetobacter spp* isolates studied [15]. Another study from North India has even reported the emergence of tigecycline and CLR *Acinetobacter baumannii* in urinary tract infections [16].

In *Pseudomonas spp* and* Acinetobacter spp.,* patient-to-patient transmission appears to play an important role. CLR Acinetobacter spp. prevalence was very less in our ICU contrary to the above studies. Stringent hand washing and surface cleaning carried out as a routine in our ICUs could likely be the reason for this. 

The MDR GNB isolates were mostly recovered from blood cultures (26.8%) followed by ulcer/surgical wound exudates (23.0%). CLR was common among MDR GNB isolated from the respiratory samples of the patients who presented with respiratory tract infection (31.4%) followed by blood samples i.e., 25% of the patients with a diagnosis of septicemia.

On the contrary, bloodstream infection by CLR MDR strain was reported as the most common infection in a study done on the emergence of CLR in MDR *Klebsiella pneumoniae* and *Escherichia coli *isolated from cancer patients, from Egypt [23]. This may be due to the neutropenic status of the cancer patients favoring GNB bloodstream infection treated with colistin. 

During treatment decisions, colistin is generally tested in MDR isolates. In this study, 54.1%, 21.8%, and 18% of the samples were from neurology ICU, medicine ICU, and neonatal ICU respectively where the patients generally stayed for a longer period of time. Other studies also mentioned that CLR in patients is related to prolonged ICU admission, which is again consistent with the use of colistin as a last resort to treat severe MDR bacterial infections like nosocomial infection [13,14]. In a study done on an enteric colonization survey, the prevalence of CLR was found to be less among enterobacterial isolates without earlier use of colistin [8].

In this study, out of the total patient data collected, 57.9% of the patients had no comorbidity, and majority of 55% of them recovered. Recovery was maximum (74.2%) among the patients with no underlying comorbidity. There were two patients with the diagnosis of brain tumors and both of them died. Patients with diabetes with underlying kidney disease were 24 in number and 83.4% of them died.

The present study showed that CLR isolates were mostly (60.4%) isolated from the samples of neurology ICU patients. Patients in neurology ICU usually stay for a prolonged period with many invasive devices and are exposed to many broad-spectrum antibiotics leading to infection with MDR strains which are again treated with colistin leading to the development of CLR variants.

Limitation of this study: Previous studies established the fact that acquired resistance to colistin by MDR GNB is transmissible and plasmid-mediated. Hence, in this study, further investigation of the plasmid gene in GNB for CLR would ascertain the definite cause of resistance. 

## Conclusions

The present study represents the CLR among the MDR isolates from a tertiary care hospital in eastern India. We found that the prevalence of CLR was high (19.6%) among all MDR GNB isolates from samples of ICU patients and that *Klebsiella pneumoniae *and* Escherichia coli *develop CLR frequently. CLR was common among the MDR strains isolated from patients with respiratory infections and samples of neurology ICU patients. Patients without any comorbid condition recovered in maximum numbers. It can be concluded that prolonged hospital stay and direct antibiotic pressure in hospitals can lead to the development of CLR variants and are considered to be nosocomial in origin. Active involvement of the microbiology department in antibiotic surveillance, as well as stewardship programs and strict infection control practices to prevent further increase in resistance to these last resorts of antimicrobials, is the need of the hour.

## References

[1] van Vught LA, Klein Klouwenberg PM, Spitoni C (2016). Incidence, risk factors, and attributable mortality of secondary infections in the intensive care unit after admission for sepsis. JAMA.

[2] Vincent JL, Rello J, Marshall J (2009). International study of the prevalence and outcomes of infection in intensive care units. JAMA.

[3] Cohen J (2013). Confronting the threat of multidrug-resistant gram-negative bacteria in critically ill patients. J Antimicrob Chemother.

[4] (2020). Countries should reduce use of colistin in animals to decrease the risk of antimicrobial resistance. http://www.ema.europa.eu/docs/en_GB/document_library/Scientific_guideline/2016/07/.

[5] Falagas ME, Kasiakou SK (2005). Colistin: the revival of polymyxins for the management of multidrug-resistant gram-negative bacterial infections. Clin Infect Dis.

[6] Schwarz S, Johnson AP (2016). Transferable resistance to colistin: a new but old threat. J Antimicrob Chemother.

[7] Wattal C, Goel N, Oberoi JK, Raveendran R, Datta S, Prasad KJ (2010). Surveillance of multidrug resistant organisms in tertiary care hospital in Delhi, India. J Assoc Physicians India.

[8] Olaitan AO, Morand S, Rolain JM (2014). Mechanisms of polymyxin resistance: acquired and intrinsic resistance in bacteria. Front Microbiol.

[9] Gales AC, Jones RN, Sader HS (2006). Global assessment of the antimicrobial activity of polymyxin B against 54,731 clinical isolates of Gram-negative bacilli: report from the SENTRY antimicrobial surveillance programme (2001-2004). Clin Microbiol Infect.

[10] Sader HS, Jones RN (2005). Antimicrobial susceptibility of uncommonly isolated non-enteric Gram-negative bacilli. Int J Antimicrob Agents.

[11] Castanheira M, Griffin MA, Deshpande LM, Mendes RE, Jones RN, Flamm RK (2016). Detection of mcr-1 among Escherichia coli clinical isolates collected worldwide as part of the SENTRY antimicrobial surveillance program. Antimicrob Agents Chemother.

[12] Bradford PA, Kazmierczak KM, Biedenbach DJ, Wise MG, Hackel M, Sahm DF (2015). Correlation of β-Lactamase production and colistin resistance among Enterobacteriaceae isolates from a global surveillance program. Antimicrob Agents Chemother.

[13] Matthaiou DK, Michalopoulos A, Rafailidis PI (2008). Risk factors associated with the isolation of colistin-resistant gram-negative bacteria: a matched case-control study. Crit Care Med.

[14] Kontopidou F, Plachouras D, Papadomichelakis E (2011). Colonization and infection by colistin-resistant gram-negative bacteria in a cohort of critically ill patients. Clin Microbiol Infect.

[15] Prim N, Rivera A, Rodríguez-Navarro J, Español M, Turbau M, Coll P, Mirelis B (2016). Detection of mcr-1 colistin resistance gene in polyclonal Escherichia coli isolates in Barcelona, Spain, 2012 to 2015. Euro Surveill.

[16] Wang Y, Tian GB, Zhang R (2017). Prevalence, risk factors, outcomes, and molecular epidemiology of mcr-1-positive Enterobacteriaceae in patients and healthy adults from China: an epidemiological and clinical study. Lancet Infect Dis.

[17] Quan J, Li X, Chen Y (2017). Prevalence of mcr-1 in Escherichia coli and Klebsiella pneumoniae recovered from bloodstream infections in China: a multicentre longitudinal study. Lancet Infect Dis.

[18] Xavier BB, Lammens C, Ruhal R, Kumar-Singh S, Butaye P, Goossens H, Malhotra-Kumar S (2016). Identification of a novel plasmid-mediated colistin-resistance gene, mcr-2, in Escherichia coli, Belgium, June 2016. Euro Surveill.

[19] (2020). European Centre for Disease Prevention and Control: antimicrobial resistance surveillance in Europe 2013. Antimicrobial resistance surveillance in Europe.

[20] (2019). Recommendations for MIC determination of colistin (polymyxin E) as recommended by the joint CLSIEUCAST Polymyxin Breakpoints Working Group. http://http//www.eucast.org.

[21] Basak S, Singh P, Rajurkar M (2016). Multidrug resistant and extensively drug resistant bacteria: a study. J Pathog.

[22] Liu YY, Wang Y, Walsh TR (2016). Emergence of plasmid-mediated colistin resistance mechanism MCR-1 in animals and human beings in China: a microbiological and molecular biological study. Lancet Infect Dis.

[23] Zafer MM, El-Mahallawy HA, Abdulhak A, Amin MA, Al-Agamy MH, Radwan HH (2019). Emergence of colistin resistance in multidrug-resistant Klebsiella pneumoniae and Escherichia coli strains isolated from cancer patients. Ann Clin Microbiol Antimicrob.

[24] Olaitan AO, Morand S, Rolain JM (2016). Emergence of colistin-resistant bacteria in humans without colistin usage: a new worry and cause for vigilance. Int J Antimicrob Agents.

[25] Lo-Ten-Foe JR, de Smet AM, Diederen BM, Kluytmans JA, van Keulen PH (2007). Comparative evaluation of the VITEK 2, disk diffusion, etest, broth microdilution, and agar dilution susceptibility testing methods for colistin in clinical isolates, including heteroresistant Enterobacter cloacae and Acinetobacter baumannii strains. Antimicrob Agents Chemother.

[26] Guérin F, Isnard C, Sinel C, Morand P, Dhalluin A, Cattoir V, Giard JC (2016). Cluster-dependent colistin hetero-resistance in Enterobacter cloacae complex. J Antimicrob Chemother.

[27] Cheong HS, Kim SY, Wi YM, Peck KR, Ko KS (2019). Colistin heteroresistance in klebsiella pneumoniae isolates and diverse mutations of PmrAB and PhoPQ in resistant subpopulations. J Clin Med.

[28] Napier BA, Band V, Burd EM, Weiss DS (2014). Colistin heteroresistance in Enterobacter cloacae is associated with cross-resistance to the host antimicrobial lysozyme. Antimicrob Agents Chemother.

